# Lumbar Puncture

**Published:** 1905-07-15

**Authors:** 


### LUMBAR PUNCTURE.

Quincke's method of puncturing the spinal theca for the purpose of investigating the state of the cerebro-spinal fluid was introduced as a therapeutic proceeding in 1891. It has unfortunately not fulfilled the expectations entertained on its behalf as a remedial measure, but continues to enjoy a considerable vogue as an aid to the diagnosis of diseases manifesting signs of cerebral and cerebro-spinal irritation. In a recently-published paper Dr. Eugene Bernstein[1] enunciates his conclusions drawn from an examination of 265 cerebro-spinal fluids at the Mount Sinai Hospital, New York, and there is much of interest in them.

Normal cerebro-spinal fluid, he says, is always absolutely colourless, supposing that no hæmorrhage accompanies the puncture. On the other hand, in cases of meningitis, whether tuberculous or purulent, some degree of opalescence is constantly present. The specific gravity ranges from 1·001 to 1·009, and the reaction of the fluid is faintly alkaline both in health and disease. The normal fluid forms no clot, nor does that derived from patients affected by tumour or abscess of the brain, by sinus thrombosis, or by hydrocephalus; while in the case of definite meningitis of whatever nature fibrin-formation is seldom absent at the expiry of six or more hours after withdrawal of the fluid. The author considers this fact an important aid to diagnosis, and quotes Wentworth to the effect that the slightest cloudiness of the fluid with the formation of a fibrin network is pathognomonic of an inflammatory exudation in the meninges. Albumin is a normal ingredient of cerebro-spinal fluid; but the quantity is very small (from 2 to 1 per mille) and an easy matter for fallacious inference from the likelihood of slight contamination with blood. As regards the reducing substance commonly considered to be pyrocatechin, which is a normal ingredient of the fluid, Dr. Bernstein endorses the following important conclusions of Comba:—

1. That in the cerebro-spinal fluid drawn during life from a healthy person there constantly exists a glucose-like reducing substance. The average quantity is 4 to 5 centigrammes to 100 c.c.

2. That in tuberculous meningitis the glucose is found in small amount at the onset, but is absent toward the end.

3. That in purulent meningitis glucose is always absent.

The author, however, qualifies his adhesion to the second of these propositions with the remark that he has never been able to demonstrate glucose at any stage of tuberculous meningitis.

Touching the cytology of the fluid, Dr. Bernstein lays stress upon the paramount necessity of excluding blood-contamination, to which particular he attributes the many discrepancies to be found in the literature. But, with all precautions, it appears that cytological inferences have little claim to be considered positive. It was at one time considered that a marked lymphocytosis was a clear index of a tuberculous meningitis, and polymorphonuclear leucocytosis of a purulent inflammation. The constancy of these associations have, however, been exploded, though it remains correct to say that the inferences have the balance of probability behind them.

Bacteriological examination has proved that there is hardly a pathogenic organism which has not at one time or another been discovered as an inhabitant of cerebro-spinal fluid in disease. By far the commonest is the intra-cellular diplococcus of Weichselbaum. Second in frequency is the tubercle-bacillus, which in the author's series of 40 cases was demonstrated no less than 38 times, figures yielding the very high percentage of 95 positive results. This degree of success Dr. Bernstein credits to technique and patience; he states that a search of several hours' duration is sometimes necessary for the discovery of the organism, and notes that the larger the series dealt with in published investigations on the subject, the higher the percentage of positive results; a fact which points strongly towards the importance of the most improved technique. The method he has employed with such marked success is as follows. If a fibrin web has formed it is removed with a sterile platinum loop, spread on a clean cover-glass, and allowed to dry; the fluid is then contrifugalised; if the quantity is large, the deposit must always be retained after each centrifugalisation, while the supernatant fluid is poured off and the tube refilled with the untreated remainder of the fluid. By this means the total deposit is collected in one tube at the conclusion of the process; a few loopfuls of this are placed upon the fibrin bed previously prepared on the cover-glass, and allowed to dry slowly before staining. With the aid of this technique the author has demonstrated tubercle bacilli in the fluid as early as 12 days before a fatal issue.

When it is considered how closely tuberculous meningitis may simulate in clinical behaviour a disease of such infinitely minor gravity as pneumonia, it becomes clear that any sound method of achieving a diagnosis deserves every recognition. Tuberculous meningitis differs from tuberculous lesions elsewhere in its rapid fatality, and thus we are denied the assistance of animal experiment in determining a diagnosis; at the same time the growth of the bacillus on artificial media is so slow that diagnosis by culture would certainly be anticipated by the death or recovery of the patient.

As to the possibility of recovery after turberculous meningitis Dr. Bernstein considers that, however rare, it is an established contingency. Four cases, which appear to be fully authenticated, are quoted from the literature, and are of sufficient interest to be here recorded.

1. A case reported by Freyhan. The patient presented the typical clinical features of tuberculous meningitis, and on two successive occasions demonstration of tubercle bacilli was achieved in the fluid derived from lumbar puncture. The patient, after an illness of several months, made a complete recovery.

2. A case reported by Henkel in 1900. A boy of ten years who recovered after an illness during which tubercle bacilli appeared in the cerebro-spinal fluid.

3. A case reported by Barth in 1902. A child of two who recovered under similar circumstances.

4. A case reported by Gross in 1902. Three punctures were made. The fluid resulting from the first showed tubercle bacilli, though the following two were negative; but the patient recovered.

A good bibliography is appended to the paper—which is in every way a convincing and valuable addition to our knowledge.

[1] Med. News, New York, June 17, 1905.

